# Effectiveness of Tunnelling Modification Technique Using Platelet-rich Fibrin and Connective Tissue Graft in Gingival Recession: A Systematic Review

**DOI:** 10.12688/f1000research.152557.1

**Published:** 2024-07-29

**Authors:** Popy Sandra, Esti Cahyani Adiati, Nurul Khairiyah, Benso Sulijaya, Yuniarti Soeroso

**Affiliations:** 1 Postgraduate Program in Periodontology, Department of Periodontology, Universitas Indonesia, Central Jakarta, Jakarta, Indonesia; 2 Department of Periodontology, Universitas Indonesia, Central Jakarta, Jakarta, Indonesia

**Keywords:** Gingival recessions, platelet-rich fibrin, connective tissue graft, tunneling technique

## Abstract

**Background:**

Gingival recession is a concern in aesthetic and functional perspective. The tunneling technique (TT) is one of the effective root coverage treatments in some gingival recession defects and is associated with favorable outcomes. This paper aims to evaluate the effectiveness of platelet-rich fibrin (PRF) and connective tissue graft (CTG) in gingival recession treatment with TT.

**Methods:**

This systematic review used the PRISMA method and electronic bibliographic searches were conducted on seven databases (Google Scholar, Wiley, Pubmed, Sage, Ovid Technologies, Quintessence Publishing, Springer) from December 2018 to January 2023. The search focused on randomized clinical trials (RCTs) that reported TT outcomes in the treatment of Miller class I and II recession with a minimum of six months follow-up.

**Results:**

Three out of 399 studies met the inclusion criteria. The three selected studies presented PRF and CTG use in multiple gingival recessions to evaluate tissue condition and clinical parameters before and after the surgical procedure. The clinical parameters evaluated were probing pocket depth (PPD), recession width (RW), width of keratinized gingiva (WKG), and vertical depth of recession (VDR). TT with PRF and TT with CTG is effective in treating gingival recession. PRF is well accepted by patients with a less invasive procedure compared to the CTG procedure. However, TT with CTG showed better results in all parameters at follow-up.

**Conclusions:**

TT with PRF can be used as an alternative to treat some gingival recession defects. However, TT with CTG produced better clinical results in recession closure.

## Introduction

Gingival recession is defined as apical migration of the gingival margin at the cemento-enamel junction (CEJ). ^1^ Gingival recession is defined as apical migration of the gingival margin at the cementoenamel junction (CEJ). ^1^ Thin biotype, less width of keratinized tissue, prominence, and root proximity make surgical therapy difficult. Many techniques and biomaterials have been developed to treat gingival recession (GR). Connective tissue graft (CTG) and coronally advanced flap (CAF) techniques are generally considered the most effective and more predictable surgical techniques for root coverage treatment with good long-term stability and are therefore declared the gold standard. ^1^ In some cases, the shortcomings of the CTG techniques is inadequate palate tissue thickness and creates a second surgical site that increases morbidity for the patient. To overcome these various disadvantages, various efforts have been made to find alternative materials for CTG. ^2^

Platelets play a crucial part in wound healing, thus utilizing platelet concentrate can help speed up the process. Growth factors (GFs) released from platelets are important for wound healing and increase cell angiogenesis, proliferation, and extracellular matrix synthesis. ^1^ Platelets have biologically active proteins, and using autologous platelet concentrates is a promising application in clinical situations that require fast healing, as in the case of tissue regeneration in periodontal plastic surgery. ^1^^,^^3^ Concentrated growth factor (CGF) is obtained by centrifuging venous blood in a special centrifuge at varying speeds (2400 to 3000 rpm). In CGF isolation, constantly modified rates can be used when obtaining platelet-rich fibrin (PRF). PRF was first developed in France for use in the field of oral and maxillofacial surgery. PRF is classified as a second-generation platelet concentrate because it is made as a natural concentrate without the addition of anticoagulants. ^1^^,^^3^

Platelet-Rich Fibrin (PRF) comprises a fibrin matrix polymerized in a tetramolecular structure, wherein platelets, leukocytes, cytokines, and circulating stem cells are incorporated. ^4^ PRF is high in leukocytes and immune cytokines, such as IL-1β, IL-4, IL-6, and TNF-α. ^5^ During wound healing, the PRF spontaneously generates a dense fibrin network, allowing for slower breakdown and, as a result, delayed release of growth factors to surrounding tissue. ^5^ The release of growth factors from PRF has been observed to last up to 7 days or longer. ^5^^–^^7^

Several studies have reported various combinations of the “tunnel” approach with CTG to treat multiple recession cases by avoiding vertical flap incisions while maintaining capillary integrity. ^1^ CTG has high predictability and good aesthetic results. This can be seen from most studies, which have predicted root coverage (RC) ranging from 69% to 97%. In addition, this technique requires a suitable donor location. ^2^^,^^8^ This systematic review aimed to evaluate the effectiveness of PRF and CTG in treating GR with the tunneling technique (TT).

## Methods

**Figure 1.**
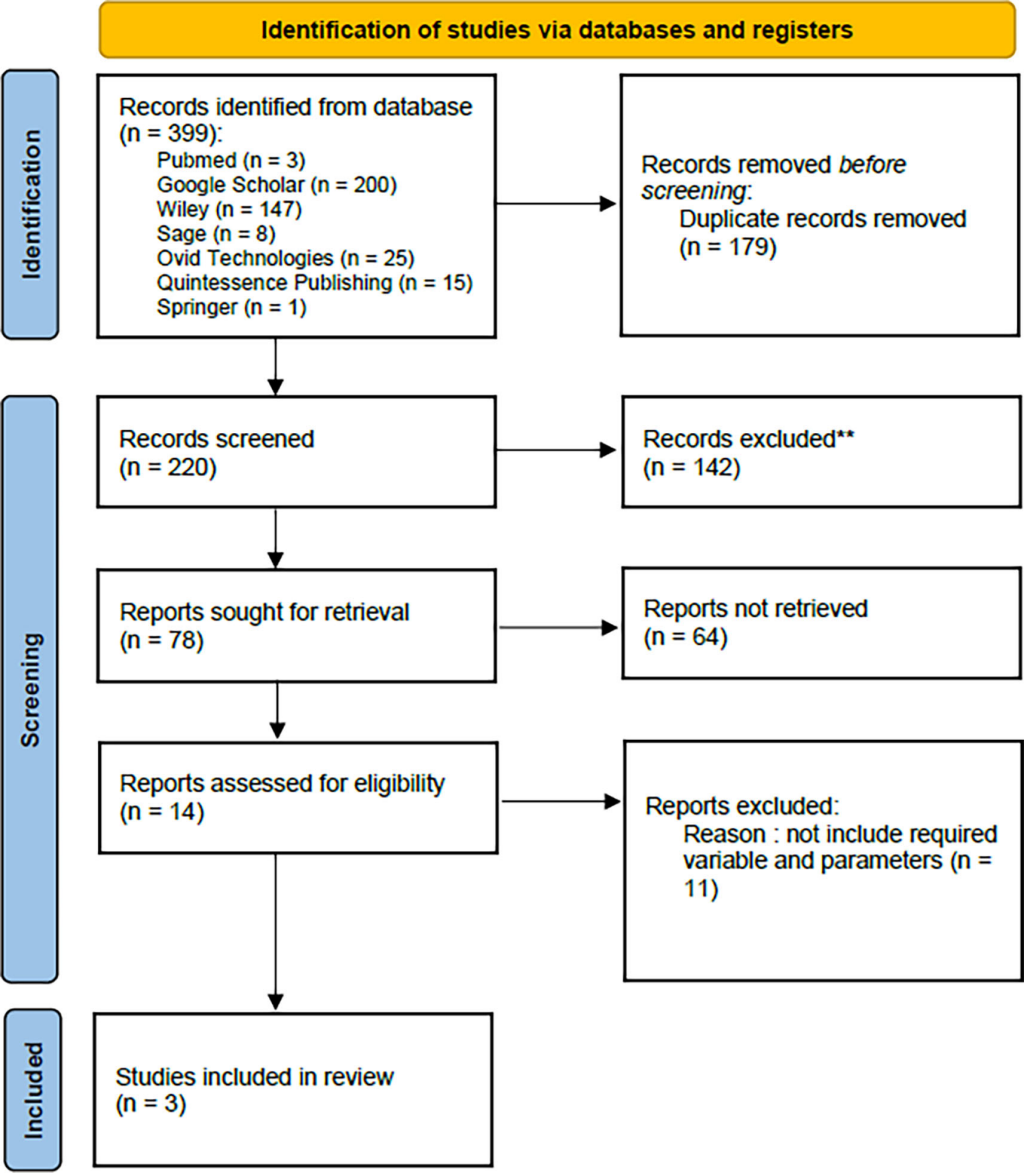
PRISMA flowchart. ^11^

This systematic review uses the PRISMA (Preferred Reporting Items for Systematic Reviews and Meta-analyses) method ( Figure 1), and specific questions are created based on the PICOS principle. ^9^^,^^10^ Research questions for the literature search are determined based on four crucial elements, namely population (P), which is patients with treatment of gingival recession, intervention (I) with tunneling technique, comparison (C) between PRF and CTG, results or outcome (O), which are the degree of epithelial attachment (DEA), the width of keratinized gingiva (WKG), and the vertical depth of recession (VDR), and study design(s) randomized clinical trial (with follow-up of at least six months after the procedure). This systematic review’s protocol has been registered with ID number CRD42024556227 in the International Prospective Register of Systematic Reviews, or PROSPERO.

In healthy adult patients who experience soft tissue recession, “what is the effectiveness of using CTG and PRF with tunneling techniques?" was the main research question for this systematic review. The following standards were used to determine which studies qualified: RCTs/CCTs study design, follow-up of at least six months after the procedure, literature comparing PRF and CTG and tunneling techniques, studies with full text in English, cases of recession in all areas (anterior/posterior, single/multiple), there is data regarding the degree of epithelial attachment (DEA), the width of keratinized gingiva (WKG), and the vertical depth of recession (VDR) in quantitative form. The focus question for this systematic review was, “In healthy adult patients who experience soft tissue recession, what is the effectiveness of using CTG and PRF with tunneling techniques?”

### Eligibility criteria

Eligible studies were included with the following criteria: RCTs/CCTs study design, minimum follow-up of 6 months after the procedure, literature comparing PRF and CTG and tunneling techniques, studies with full text in English, cases of recession in all areas (anterior/posterior, single/multiple), there is data regarding the degree of epithelial attachment (DEA), the width of keratinized gingiva (WKG), and the vertical depth of recession (VDR) in quantitative form.

### Search

Using a particular search strategy (Tunnelling Technique AND Platelet Rich Fibrin AND Connective Tissue Graft AND Gingival Recessions), an electronic search was conducted on seven databases (Google Scholar, Wiley, Pubmed, Sage, Ovid Technologies, Quintessence Publishing, Springer). A literature search was done to discover English-language publications from 2018 to January 2023. The entire systematic review approach was carried out by four people, by performed a methodical examination across the databases. After deleting duplicate documents, the authors manually reviewed the abstracts and titles. After that, the authors assessed full-text publications to choose the research that satisfied the inclusion requirements. If consensus was not reached, experienced supervisors were consulted for the final decision.

### Synthesis of results

Data were extracted from the accompanying full-text articles by the authors using an Excel Spreadsheet (Microsoft) created specifically for this review. The studies was then assessed for the risk of bias using JBI tools. Data extracted included the author’s name, study design, number of patients and number of implants, patient age, implant location, type of soft tissue defect, soft tissue augmentation technique performed, time of procedure follow-up time, and outcomes. During this process, any discrepancies in the review will go through a discussion process between the authors.

## Results

The search on seven electronic databases using the supplied keywords yielded a total of 399 papers. More specifically, 3 studies were acquired from PubMed, 200 from Google Scholar, 147 from Wiley, 8 from Sage, 25 from Ovid Technologies, 15 from Quintessence Publishing, and 1 from Springer. The duplication was eliminated carefully using Excel, obtaining 179 repeated studies. A total of 220 studies passed title and abstract screening, with 142 removed due to noncompliance with the author’s specified inclusion and exclusion criteria. As a result, only fourteen research remain to be analyzed. From 14 studies, there are 11 studies that are not include the required parameters and variable. Only three studies met the inclusion criterias ( Tables 1 and 2).

**Table 1.** Basic information about the included studies.

No	Author	Year	Type *of Study*	Sample Size	Sample Groups	Technique	Recession Type	*Follow Up*	Parameter	Conclusion
1	S Hegde *et al.* ^2^	2021	*Randomized clinical study*	32 defects in 10 patients	1. *VISTA* + *PRF* 2. *VISTA* + *CTG*	*VISTA*	Miller Class I or II	Six months after surgery	*PI, GI, PPD, RAL, RD, R, WKG, %RC*	• *VISTA combined with CTG or PRF (Miller Class I and Class II recession) => predictable results.* • *Significant improvement in clinical parameters from baseline to 6 months in both groups.* • *CTG is the gold standard. PRF can be used as an alternative*
2	BV Subbareddy *et al.* ^17^	2020	*Randomized prospective clinical comparative study*	20 patients with multiple gingival recession	1. 10 test group subjects (VISTA with PRF) 2. 10 control groups (VISTA with SCTG).	Vestibular incision subperiosteal tunnel access (VISTA) technique	Miller Class I or II	Early, three months, and six months	*PI,GI, PD,CAL, RW, RD,KT*, and *GT*	• (VISTA with PRF) and (VISTA with SCTG), resulting in varying root coverage. • VISTA + SCTG = superior to VISTA +PRF in all parameters • VISTA +PRF and VISTA + SCTG stable results • Both techniques provide excellent color • VISTA + PRF can be used if the sole objective is to obtain root coverage, provided there is an adequate apical zone of KT
3	V Chandra *et al.* ^20^	2023	*Randomized clinical study*	17 patients (aged 26–47 years) with 40 defect locations	1. 20 defect sites (9 patients) treated +PRF (Group I) 2. 20 defect locations (8 patients) + CTG (Group II).	*Pouch and Tunnel Technique*	Miller Class I or II	*1-, 3-, and 6-months*	*PI, GI, PPD, CAL, HGR, VGR, WAG, WKG, GTMB,* dan *GTIP*	• Pouch and tunnel procedure + PRF or CTG is effective for RC • Limitations The results achieved on-site with PRF are comparable to CTG (Gold standard) => better patient acceptability and less invasive approach • Long-term multicenter randomized controlled clinical trials may be needed to evaluate clinical outcomes for autologous PRF: CTG with pouch and tunnel.

**Table 2.** Results of included studies.

No	*Author (Year)*	Sample Size	*Follow up*	Group	MEASURED PARAMETERS (mm)																							
					*PPD*				*RD/VGR*				*RW/HGR*				*WKG/KTW*				*Gingival Thickness/GTMB*				*%RC*			
					*baseline*	*3-6 months*	*mean 6m*	*P value*	*Baseline*	*Six months*	*mean 6m*	*P value*	*Baseline*	*Six months*	*mean 6m*	*P value*	*baseline*	*Six months*	*mean 6m*	*P value*	*baseline*	*Six months*	*mean 6m*	*P value*	*percentage of RC*			*P value*
1	S Hegde *et al.* (2021) ^2^	32 defects in 10 patients	Six months	Group 1 VISTA + PRF	*NA*	*NA*	1.31±0.60	*NA significant improvement*	2.563±0.964	0.813±1.560	2.844	*Baseline (P = 1), 6 months (P=0.756 (NS)*	*NA*	*NA*	R1.188±1.642	*NA significant improvement*	3.500±0.873	5.875±1.784	2.438±0.964 dan 5.125±0.885	*<0.001 (P = 0.284) not statistically significant*	*NA*	*NA*	*NA*	*NA*	*Mean* dan *SD* 83.250±25.027			P=0.401 *(NS)*
				Group 2 VISTA + CTG	*NA*	*NA*	1.37±0.5		2.563±0.965	0.500±0.816	3.598		*NA*	*NA*	1.063±1.642		2.438±0.964	5.125±0.885	2.687±0.946	<0.001 (VHS)	*NA*	*NA*	*NA*	*NA*	*Mean* dan *SD* 86.438±22.798			,
2	BV Subbareddy *et al.* (2020) ^17^	20 patients with multiple gingival recession	early, three months and six months	Group 1 (test) VISTA + PRF	2.65±0.56	2.15±0.33	2.14±0.39	0,878	1.62±1.34	3.24±1.19	1.69±1.59	0.000*	3.87±1.13	2.36±1.91	2.27±1.87	0.010*	2.03±0.75	2.90±0.96	2.90±0.99	0.000*	0.80±0.13	3.64±0.97	1.09±0.27	0.020*	33 recessions=> 10 recessions (30.3%) complete root coverage, 23 69.67% partial closure.			
				Group 2 (control) VISTA *+ SCTG*	2.44±0.31	2.13±0.24	2.10±0.26		2.11±0.32	2.74±0.72	1.78±0.30		2.75±0.72	2.11±0.32	1.78±0.30		2.03±0.75	3.64±0.97	3.74±1.00		0.80±0.10	1.02±0.11	0.99±0.08		25 recessions, 15 recessions (60%) completely closed 10 (40%) tertutup sebagian			
3	V Chandra, *et al.* (2022) ^20^	17 patients	*1-, 3-, and 6-month posttreatment.*	Grup 1: *pouch and tunnel* + *PRF*	1.75±0.14	0.95±0.05	0.00	1.000	2.10±0.24	0.65±0.20	0.20	0.533	2.90±0.28	0.80±0.22	0.15	911	3.85±0.31	5.30±0.26	0.30	0.813	2.00±0.10	2.95±0.05	0.05	0.873	*NA*	*NA*	*NA*	*NA*
				Grup 2: *pouch and tunnel + CTG*	1.60±0.11	0.95±0.05			2.30±0.21	0.85±0.25			2.75±0.18	0.95±0.29			3.65±0.38	5.00±0.40			2.10±0.07	2.90±0.07			*NA*	*NA*	*NA*	*NA*

Abbreviations: *plaque index (PI), gingival index (GI), probing probing pocket depth (PPD), relative attachment level (RAL), recession depth (RD), recession width (RW), width of keratinized gingiva (WKG), percentage of root coverage (%RC), Subepithelial Connective Tissue Graft (SCTG), vestibular incision subperiosteal tunnel access (VIST)), platelet-rich fibrin (PRF), connective tissue graft (CTG)*.

The risk of bias evaluation for the randomized clinical trial was carried out using the Joanna Briggs Institute (JBI) tools, which contains thirteen domains. The JBI is a reliable and valid tool for assessing the likelihood of bias in studies. Each study’s quality was evaluated independently by two reviewers. The JBI score for each study was computed using the checklist, and the results were presented as percentages. The study with a JBI score of 20-49% was considered high risk of bias, whereas those with 50-79% and 80-100% were moderate and low risk of bias, respectively. Table 3 shows the results of the risk of bias evaluations.

**Table 3.** Risk of Bias Assesment of Selected Studies with JBI tools.

Study	Study Type	Q1	Q2	Q3	Q4	Q5	Q6	Q7	Q8	Q9	Q 10	Q 11	Q12	Q13	Scores	*Risk of Bias Criteria*
S Hegde *et al.* ^2^	*RCTs*	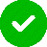	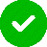	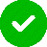	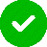	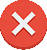	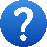	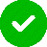	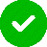	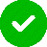	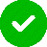	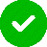	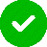	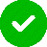	84.6%	Low risk
BV Subbareddy *et al.* ^17^	*RCTs*	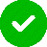	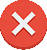	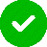	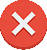	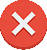	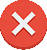	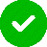	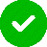	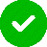	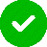	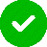	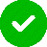	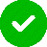	69.2%	Moderate
V Chandra *et al.* ^20^	*RCTs*	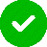	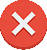	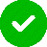	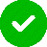	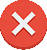	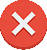	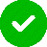	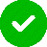	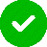	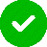	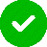	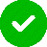	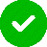	76.9%	Moderate

**Abbreviations:** JBI = Joanna Briggs Institute; 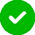 Yes; 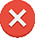 No; 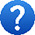 Uncertain; Q1: Was true randomization used for assignment of participants to treatment groups?; Q2: Was allocation to treatment groups concealed?; Q3: Were treatment groups similar at the baseline?; Q4: Were participants blind to treatment assignment?; Q5: Were those delivering treatment blind to treatment assignment?; Q6: Were outcomes assessors blind to treatment assignment?; Q7: Were treatment groups treated identically other than the intervention of interest?; Q8: Was follow up complete and if not, were differences between groups in terms of their follow up adequately described and analyzed?; Q9: Were participants analyzed in the groups to which they were randomized?; Q10: Were outcomes measured in the same way for treatment groups?; Q11: Were outcomes measured in a reliable way?; Q12: Was appropriate statistical analysis used?; Q13: Was the trial design appropriate, and any deviations from the standard RCT design (individual randomization, parallel groups) accounted for in the conduct and analysis of the trial?

## Discussion

Gingival recession is defined as abnormal apical displacement of the gingival margin in relation to the cemento-enamel junction (CEJ), exposing the root surface. This causes functional and aesthetic issues, such as dentin hypersensitivity accompanied by open, localized, or widespread root surfaces, and is a component of mucogingival deformity, an anatomical defect caused by a variety of etiological factors. ^11^^–^^13^

The goal of regenerative treatment, in addition to addressing aesthetic concerns, is to ensure the stability of soft tissue attachment. According to the research by Zucchelli et al., long-term maintenance of treatment results can be achieved by focusing on the quality of epithelial attachment and enhancing soft tissue volume. ^11^^,^^12^

CTG is considered as the gold standard for treating soft tissue defects. ^14^ Anatomically and histologically, the area around the first premolar contains the most fat and glandular tissue, offering substantial volume when considering tissue quantity. The section of the palate from the second premolar to the second molar has more collagen and less fatty tissue and glands, making it an ideal donor site for grafts in the treatment of gingival recession. ^15^ The limitations of CTG include the need for a second surgical site, the thickness of the palatal mucosa, and the shape of the palate. Consequently, alternatives to the Subepithelial Connective Tissue Graft (SCTG) have been explored, such as the acellular dermal matrix (ADM) and collagen membrane. However, using ADM as a substitute for SCTG increases treatment costs, making it less accessible for some patients. Additionally, the source of these biomaterials (typically from cattle or pigs) may be unacceptable to many patients due to religious restrictions. Therefore, SCTG remains the most recommended option for periodontal plastic surgery procedures. ^16^

Covering multiple root recessions presents a greater challenge in periodontitis treatment. ^17^ Among the surgical techniques described in the literature for treating gingival recession are the tunneling technique (TT) and coronally repositioned flap (CAF), which consider the integrity of the interdental papilla. The SCTG technique has demonstrated a high success rate in patients with multiple recessions. ^18^ The pouch and tunneling technique has been widely used and shown varying degrees of success. ^17^

**Figure 2.**
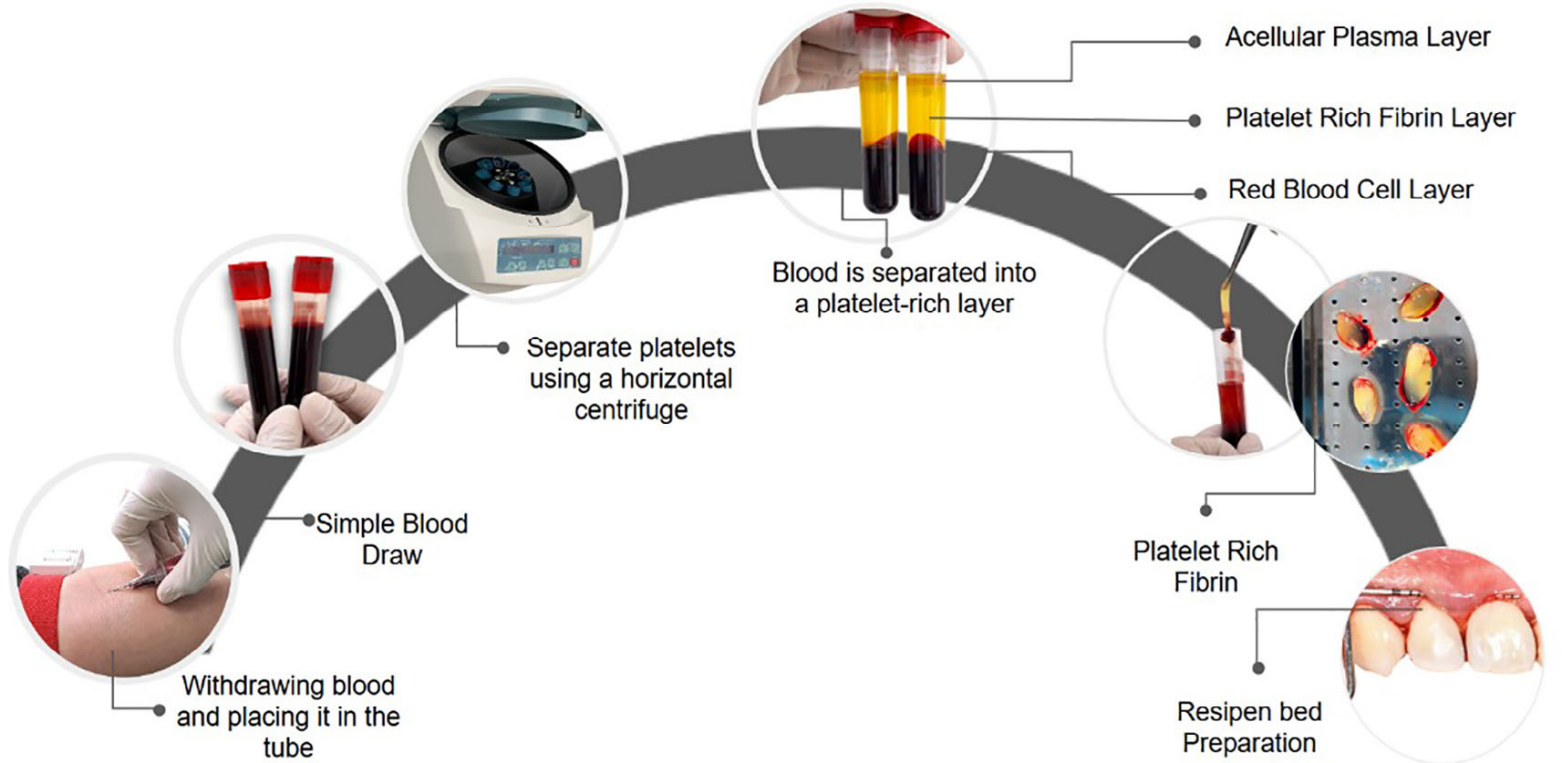
PRF Preparation Method for Tunneling Technique. Source: Own representation based on Mohan et al, 2019.

As a surgical adjuvant, platelet concentrate (PRF) effectively promotes soft tissue healing, facilitates wound closure, and enhances aesthetic outcomes. ^19^ PRF obtained from the same patients has been shown to contain numerous growth factors and has been successfully used in various surgical procedures. ^19^ Because PRF is not derived from another species, it is cost-effective, easy to prepare, and well-accepted by patients. PRF was obtained following the approach recommended by Choukroun *et al.* No biochemical blood manipulation is required, and neither bovine thrombin nor anticoagulants are needed prior to surgery. To obtain PRF Figure 2, 20 milliliters of venous blood are drawn from the patient via antecubital vein venipuncture and placed in two sterile 10 ml glass test tubes. These tubes are immediately centrifuged at 3000 rpm for 10 minutes, resulting in the formation of a PRF membrane. ^17^^,^^19^^,^^20^

The primary goal of this systematic review is to assess the effectiveness of using the TT technique in combination with CTG and PRF. The use of the pouch and tunnel approach alongside CTG or PRF for treating GR has not yet been extensively compared in the literature. ^20^ An electronic search conducted over the past five years identified only three studies meeting the inclusion criteria, which involved the simultaneous use of TT + CTG and TT + PRF. This highlights the necessity for long-term investigations. ^20^ Research by Chandra *et al.* comparing the regenerative potential of autologous PRF and autogenous CTG on patient satisfaction in terms of postoperative discomfort, reduced root sensitivity, and improved esthetics. ^16^^,^^20^

This study is the first randomized controlled trial conducted to equate the clinical effectiveness of PRF or CTG with the pouch technique and tunneling for the management of GR. ^20^ Chandra *et al.* stated that the pouch and tunneling + PRF or pouch and tunneling + CTG procedure was effective for RC. ^16^^,^^20^

The limited results obtained from using TT + CTG make PRF is considered to be comparable to CTG (gold standard). This is supported by the evidence that PRF has better patient acceptance and is a less invasive approach. However, long-term multicenter randomized controlled clinical trials may be needed to evaluate the clinical outcomes of the pouch and tunneling technique + autologous PRF compared with the pouch and tunneling technique + CTG. ^20^ The scant evidence presented contrasts with the study conducted by Abu-Ta’a et al., which assessed and compared the clinical outcomes of advanced-platelet-rich fibrin (A-PRF) and CTG in treating Miller class I/II gingival recession in marginal tissues. ^21^ Tested intergroup analysis showed statistically significant differences in recession parameters between groups at 3 and 6 months, with results that were better for the CTG group. ^21^ This study demonstrated that A-PRF and CTG effectively treated gingival recession defects. However, CTG produces better clinical results in reduced recession height and width. ^21^

PRF cannot function as a direct alternative biomaterial like CTG, which is the gold standard. PRF can be applied in cases with contraindications to CTG harvesting of the palate where the patient and physician wish to reduce morbidity and are willing to accept less than optimal results. ^1^^,^^2^ PRF does not require a second donor site, is cost-effective, and prevents graft site complications. However, PRF did not improve root coverage as much as CTG in GR Miller classes I and II. These findings are not enough to reveal the actual clinical effect of TT + PRF on the treatment of GR. More studies are needed to investigate and compare the effectiveness of PRF in terms of root coverage. ^1^

Hedge *et al.* stated that although excellent aesthetic results of CTG have been achieved with root coverage occurring in between 69% and 97% of cases, this technique requires a suitable donor site. ^2^ Within the limitations of this systematic review, it can be concluded that CTG still shows long-term results (12–18 months) were superior to ADM regardless of flap design or surgical technique. When CTG retrieval is not indicated, ADM can be a good alternative in treating gingival recession. Further studies with longer follow-up are needed to determine the long-term stability of xenogeneic and allogeneic soft tissue grafts. ^10^

Subbareddy *et al.* analyzed the VISTA technique with PRF and VISTA with SCTG and obtained various RC results. ^17^ VISTA applied with SCTG showed superior results to VISTA with PRF in all parameters. VISTA with PRF and VISTA with SCTG both show stable results and provide excellent color results. ^17^ VISTA with PRF can be used if the sole aim is to obtain root coverage provided an adequate Keratinized Tissue (KT) apical zone. ^17^ The studies discussed in this literature state the effectiveness of using TT + CTG and TT + PRF for various dental procedures and periodontal. Jankovic *et al.* and Aroca *et al.* found that the combination of TT + CTG and TT + PRF resulted in significant improvements in clinical parameters, such as reduced gingival recession and increased keratinized tissue width. Jankovic *et al.* stated that using TT + CTG and TT + PRF is an effective approach for soft tissue augmentation and root coverage procedures. ^10^^,^^21^ Further research is necessary to assess the long-term stability and success rate of these techniques. ^22^^,^^23^

## Conclusions

Modern methods, such as CTG and bio-mediators, are anticipated to deliver both aesthetic and functional outcomes in the regeneration of periodontal tissue. The results achieved using both methods show that they are highly successful and can be applied in the treatment of RC. Despite the observed statistical differences, the PRF method is equally suitable as an alternative to CTG for the treatment of RC, given its advantages in patient comfort and simplicity of implementation. The primary statistical difference between the two methods is the amount of keratinized gingiva obtained, which can be related to the histological structure of the soft tissue graft. A wider zone of keratinized gingiva may influence the long-term stability of the results achieved. Although TT + PRF can be used as an alternative to treat some GR defects, TT + CTG produces better clinical results regarding reduced recession height and width. The limitation of this study is that research comparing the use of TT with CTG and TT with PRF is still very limited. PRF does not increase root coverage as much as CTG, so more studies are needed to analyse and compare the effectiveness of TT with CTG and TT with PRF in root coverage

## Ethics and consent

Ethical approval and consent were not required

## Data Availability

Figshare: Checklist for Effectiveness of tunnelling modification technique using platelet-rich fibrin and connective tissue graft in gingival recession: a systematic review, DOI: 10.6084/m9.figshare.26005375.v1. ^24^ This project contains the following underlying data:
- PRISMA checklist.docx Figshare: Figure 1. Prisma Diagram of Effectiveness of tunnelling modification technique using platelet-rich fibrin and connective tissue graft in gingival recession: a systematic review, DOI: 10.6084/m9.figshare.26068684. ^25^ This project contains the following und erlying data:
- PRISMA flowchart.docx Data are available under the terms of the Creative Commons Zero “Universal” data waiver (CC0 1.0 Public domain dedication).
