# Supplementary material for: Effectiveness of Tunnelling Modification Technique Using Platelet-rich Fibrin and Connective Tissue Graft in Gingival Recession: A Systematic Review
Source: F1000Res. 2024 Jul 29;13:842. [Version 1] doi: 10.12688/f1000research.152557.1 (PMC12538278; doi:10.12688/f1000research.152557.1)
Supplement: Peer review history

# Reviewer report: Reviewer response for version 1

**DOI:** 10.5256/f1000research.167334.r320340

**Published:** 2024-10-30

**Authors:** Mustafa Tunali

**Affiliations:** 1 Çanakkale Onsekiz Mart University, Çanakkale, Turkey

Although it is a systematic review written on a current topic, there are important deficiencies:

 1. First of all, an attempt was made to compile studies comparing the clinical use of PRF and CTG in the treatment of gingival recession. However, since Tunnelling Modification Technique was also included as a surgical technique requirement, only 3 studies were able to be included in the systematic review. The success of Tunnelling Modification Technique can be evaluated separately with studies comparing different surgical techniques using the same material. This may be a separate systematic review topic.

 2. The second important deficiency is that all PRFs are put in the same category without being fully explained. In addition, material thickness, PRF type, and many important factors are not discussed along with surgical technique. This greatly reduces the power of the systematic review.

 If these important issues are taken into consideration, all these deficiencies are eliminated, and it can be designed to include more than 10 studies, it can be a good systematic review.

Are the rationale for, and objectives of, the Systematic Review clearly stated?

Partly

Is the statistical analysis and its interpretation appropriate?

Not applicable

If this is a Living Systematic Review, is the ‘living’ method appropriate and is the search schedule clearly defined and justified? (‘Living Systematic Review’ or a variation of this term should be included in the title.)

Yes

Are sufficient details of the methods and analysis provided to allow replication by others?

Partly

Are the conclusions drawn adequately supported by the results presented in the review?

Partly

Reviewer Expertise:

periodontology

I confirm that I have read this submission and believe that I have an appropriate level of expertise to confirm that it is of an acceptable scientific standard, however I have significant reservations, as outlined above.

# Reviewer report: Reviewer response for version 1

**DOI:** 10.5256/f1000research.167334.r320342

**Published:** 2024-10-23

**Authors:** Shiva Shankar Gummaluri

**Affiliations:** 1 GITAM Dental College and Hospital, Visakhapatnam, India

Present study was a well framed systematic review. Entire structure was made systematically, PRISMA guidelines were followed, search strategies were perfectly executed. Risk of Bias was well explained. Tables were wwll explained. Discussion was well explained and studies were well quoted with updated knowledge. Present systematic review also gave a proper conclusion after sequential analytical steps

Are the rationale for, and objectives of, the Systematic Review clearly stated?

Yes

Is the statistical analysis and its interpretation appropriate?

Yes

If this is a Living Systematic Review, is the ‘living’ method appropriate and is the search schedule clearly defined and justified? (‘Living Systematic Review’ or a variation of this term should be included in the title.)

Yes

Are sufficient details of the methods and analysis provided to allow replication by others?

Yes

Are the conclusions drawn adequately supported by the results presented in the review?

Yes

Reviewer Expertise:

Periodontology, Implantology, Platelet Concentrates, Periodontal Regeneration

I confirm that I have read this submission and believe that I have an appropriate level of expertise to confirm that it is of an acceptable scientific standard.

# Reviewer report: Reviewer response for version 1

**DOI:** 10.5256/f1000research.167334.r313660

**Published:** 2024-09-11

**Authors:** Triveni M Gowda

**Affiliations:** 1 Bapuji Dental College and Hospital,, Davanagere - 557 004, Karnataka, India

1. Overall the article is fairly well written and the topic of research is quite interesting

 2. General grammatical errors needs to be corrected.

 3. For a review article the number of references taken seem to be low.

 4. Overall the major issue that this particular systematic review faces are that the authors have not been able to retrieve 90% of the shortlisted articles. This not only limits the exclusion of quality literature but also limits the scope of systematic reviews only to freely available literature.

 5. All the three selected studies have almost a very similar study population as well as sample size. The population on which the studies have been conducted seems to be quite homogeneous and inclusion of only 3 studies that too on the same population for a systematic review does not same to be ideal.

 6. Reviewer would suggest the authors to try to retrieve as much as possible of the 64 articles which were not available and include those to do more thorough review of the available literature.

**ABSTRACT**

 7. The ‘tunnelling technique’ line is underexplained, and vaguely presented provide appropriate technique.

 8. In the background, specify the type of gingival recession.

 9. In the result section – “PRF is well accepted by patients with a less invasive procedure compared to the CTG procedure” can be further improved and modified.

 10. In the conclusion section – ‘TT with CTG produced better clinical results in recession closure’. Provide a detailed explanation of how it is better with respect to parameters.

**INTRODUCTION**

 11. Definition of the gingival recession is repeated. Rectify the same.

 12. Mention as ‘root’ prominence in place of prominence.

 11. Provide and highlight additional shortcomings of CTG.

 13. Specify the isolation rate of CGF.

 14. Specify the duration range of PRF in accordance with the most recent articles.

**METHODS**

 15. Provide the reference where the ‘degree of epithelial attachment (DEA)’ is considered as an outcome measure for tunnelling procedure in gingival recession procedure.

 16. Only 3 reference articles have been provided and VISTA alone has been taken as reference. Justify

 17. Correct the spelling of ‘recipient’ in the Figure 2.

**DISCUSSION**

 18. Shape of the palate is not a direct limitation. Justify the same.

 19. Mention “coronally repositioned flap (CAF)” as coronally advanced flap.

 19. How PRF enhances esthetic outcomes. Justify.

 20. Long term studies are available for the tunnelling procedures. Add the same in the discussion part.

 21. Additional factors aiding for gingival thickness are not mentioned.

 22. Why ADM studies are considered for this review /added. Justify?

Are the rationale for, and objectives of, the Systematic Review clearly stated?

Partly

Is the statistical analysis and its interpretation appropriate?

Partly

If this is a Living Systematic Review, is the ‘living’ method appropriate and is the search schedule clearly defined and justified? (‘Living Systematic Review’ or a variation of this term should be included in the title.)

No

Are sufficient details of the methods and analysis provided to allow replication by others?

No

Are the conclusions drawn adequately supported by the results presented in the review?

Partly

Reviewer Expertise:

Inter-relationship of periodontitis with systemic disease, Laser, Photo-biomodulation, Platelet Rich Fibrin, Osseous defects, gingival aesthetics, minimal invasive surgical procedures

I confirm that I have read this submission and believe that I have an appropriate level of expertise to confirm that it is of an acceptable scientific standard, however I have significant reservations, as outlined above.
